# A Review of Recent Advances in Research on PM_2.5_ in China

**DOI:** 10.3390/ijerph15030438

**Published:** 2018-03-02

**Authors:** Yaolin Lin, Jiale Zou, Wei Yang, Chun-Qing Li

**Affiliations:** 1 College of Mechanical Engineering, Shanghai University of Engineering Science, 333 Longteng Road, Shanghai 201620, China; 2 School of Civil Engineering and Architecture, Wuhan University of Technology, Wuhan 430070, China; trista@whut.edu.cn; 3 College of Engineering and Science, Victoria University, Melbourne 8001, Australia; Wei.Yang@vu.edu.au; 4 School of Engineering, Royal Melbourne Institute of Technology, Melbourne 3000, Australia; chunqing.li@rmit.edu.au

**Keywords:** PM_2.5_, China, impact, I/O relationship, control

## Abstract

PM_2.5_ pollution has become a severe problem in China due to rapid industrialization and high energy consumption. It can cause increases in the incidence of various respiratory diseases and resident mortality rates, as well as increase in the energy consumption in heating, ventilation, and air conditioning (HVAC) systems due to the need for air purification. This paper reviews and studies the sources of indoor and outdoor PM_2.5_, the impact of PM_2.5_ pollution on atmospheric visibility, occupational health, and occupants’ behaviors. This paper also presents current pollution status in China, the relationship between indoor and outdoor PM_2.5_, and control of indoor PM_2.5_, and finally presents analysis and suggestions for future research.

## 1. Introduction

**Figure 1:**
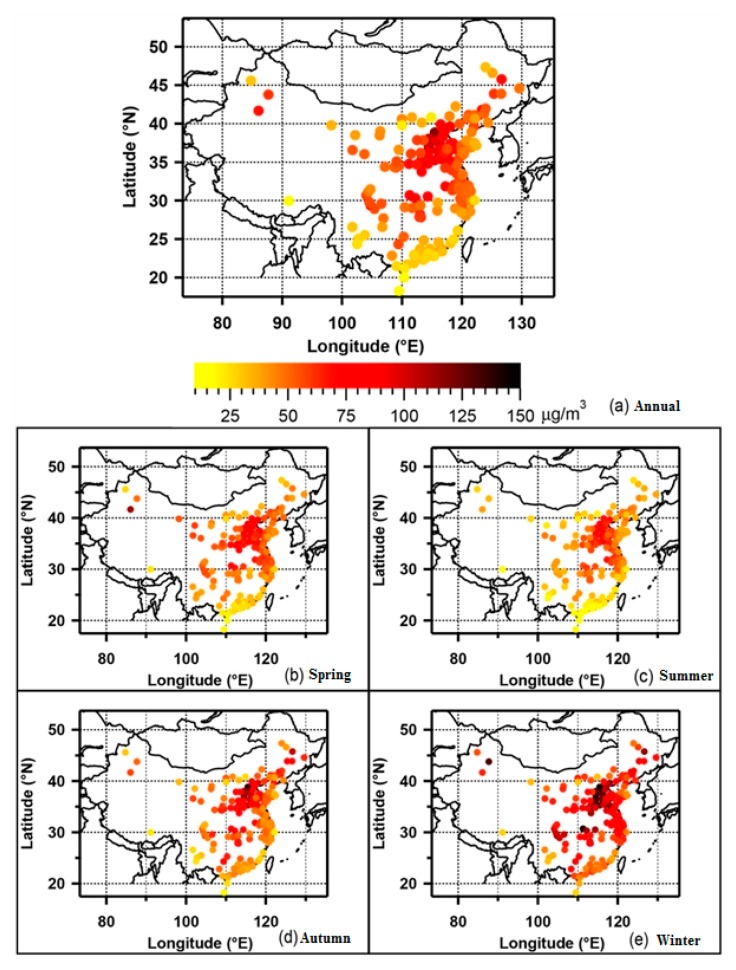
Spatial and temporal distribution of PM_2.5_ in Chinese cities [5].

In recent years, hazy weather caused by multiple pollutants, with PM_10_ (cutoff sizes ≤ 10 µm, inhalable particles) and PM_2.5_ (cutoff sizes ≤ 2.5 µm, particles that can enter the lungs) as the main pollutants, has affected large areas of China, lasting for a long time. It has a significant regional characteristic, which is shown in Figure 1 [1]. According to the data collected from the air quality monitoring stations in 338 big cities in China, the range of annual average concentrations of PM_2.5_ in 2015 in the 388 cities was 11–125 μg·m^−3^ with an average value of 50 μg·m^−3^. PM_2.5_ was the primary pollutant for 66.8% of the severely polluted days. In 2016, the average annual concentration of PM_2.5_ was 12–158 μg·m^−3^, with an average value of 47 μg·m^−3^, and PM_2.5_ was the main pollutant for more than 80.3% of the days with severe pollution [2,3]. PM_2.5_ has thus become the primary pollutant of atmospheric particulate pollution in China [4]. Compared with the coarser particles, PM_2.5_ is smaller in size, larger in surface area, and more easily transported, which implies more toxicity and harmful substances that can penetrate deep into the human body. PM_2.5_ can stay in the atmosphere for a long time and travel for a long distance. Therefore, it has a greater impact on human health and the quality of the atmospheric environment. It has always been a hot topic in various related research fields around the world.

Since the 1980s, the USA and some European countries have conducted extensive studies on PM_2.5_, which are mainly related to the spatial and temporal distribution of PM_2.5_ concentrations, emission inventory, emission characteristics, source analysis and impact of PM_2.5_ on atmospheric visibility and human health [6]. In 1997, USA took the lead in establishing environmental air quality standards for PM_2.5_ and specified that the high limit of annual average PM_2.5_ concentration is 15 μg·m^−3^, and the 24 h concentration limit is 65 μg·m^−3^.

**Figure 2:**
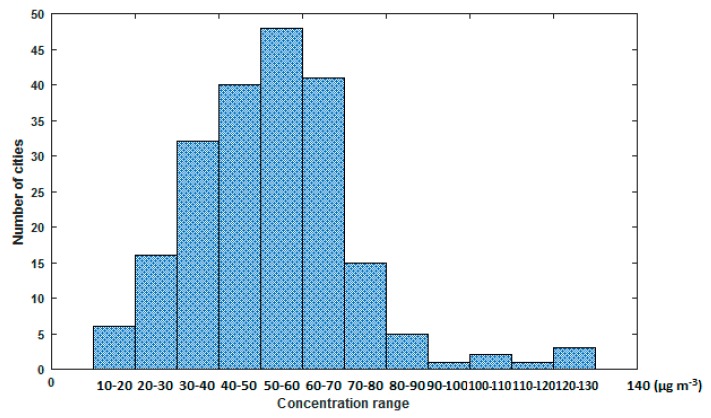
Histogram distribution of PM_2.5_ concentration [7].

Two revisions have been made since then. Other countries (organizations) have also set PM_2.5_ concentration limits of their own (see Table 1). In China, it was not until in 2012 when the current ambient air quality standard was established and the concentration limit of PM_2.5_ was incorporated into the standard. The standard adopts the maximum limits set by the World Health Organization (WHO), i.e., the annual average concentration limit is 15 μg·m^−3^, and the 24-h concentration limit is 35 μg·m^−3^. However, it was not enforced nationwide until 2016. According to the latest PM_2.5_ concentration data published by WHO on 17 April 2016, the annual average PM_2.5_ concentration among 210 cities in China was in the range of 11–128 μg·m^−3^ [7]. It is noted that only 1.4% of the cities were able to meet the first level standard in China. The histogram distribution of PM_2.5_ concentrations is shown in Figure 2 [7]. It can be concluded that the problem of PM_2.5_ pollution in China is very serious and it is urgent to take action to control PM_2.5_ emission without delay.

Due to the lack of long-term and large-scale monitoring data, compared with developed countries, research on PM_2.5_ in China started late and can be divided into three stages. The first stage was before 2004, and at the time the research on PM_2.5_ was of small scale and tentative. The studies were conducted mainly in major cities such as Beijing, Guangzhou, Nanjing and Shanghai. There were few studies on small and medium-sized cities, but only simple statistical and principle analysis on the data had been carried out [8,9,10,11,12,13,14]. For example, Wu et al. [16] conducted two-year data monitoring on PM_2.5_ concentration in Guangzhou, Wuhan, Lanzhou and Chongqing, and found that the PM_2.5_ concentration in the atmosphere generally exceeded 2–8 times of the limit set by the U.S. standard. Wang et al. [17] collected and analyzed nearly 10-year PM_2.5_ concentration data in urban and clean areas in China, and concluded that PM_2.5_ pollution is heavy in most parts of China. At the same time, He et al. [18] also collected PM_2.5_ concentration data from July 1997 to September 2000 in the city center and urban area in Beijing. It was shown that the seasonal variation of PM_2.5_ concentration was remarkable, with the highest in winter and the lowest in summer. Yang et al. [19] set up PM_2.5_ sampling points in Chegongzhuang and Tsinghua University in Beijing and started to discuss on the chemical composition characteristics of PM_2.5_. Huang et al. [20] collected 50 samples in five typical urban function areas of Nanjing in winter, spring and autumn and analyzed the PM_2.5_ pollution level. Wang et al. [21] studied the PM_2.5_ concentration in spring in Nanjing. Yang et al. [22] started to consider the source of PM_2.5_ in the atmosphere in Beijing.

The second stage is from 2004 to 2011. Although the research areas on PM_2.5_ gradually expanded, overall the research was still relatively straightforward, which were mainly related to the toxic effects of PM_2.5_ on cells [23,24,25,26,27,28], source analysis [29,30,31,32,33,34], and chemical composition analysis [35,36,37,38,39,40,41,42], etc. The third stage is from 2012 till now, due to the establishment of China’s PM_2.5_ air quality standards and gradual developments of nationwide PM_2.5_ observation stations, the number of researches on PM_2.5_ have increased exponentially. Since then, more and more disciplines have become involved in the study on PM_2.5_, but overall the research still lags behind, compared with developed countries. This paper aims at studying the advances in PM_2.5_ on research in China in recent years from the following four aspects: the sources of PM_2.5_, the influence of PM_2.5_, the correlation of indoor and outdoor PM_2.5_ concentration and the control of PM_2.5_, and trying to explore new insights for the scholars of future research.

## 2. Sources of PM_2.5_

### 2.1. Sources of PM_2.5_ in Urban Atmosphere

The sources of PM_2.5_ in urban atmosphere are very complicated. They can mainly be categorized into primary and secondary sources, of which primary sources refer to the direct emissions of various sources such as combustion sources. The secondary sources come from particles generated from the chemical processes in the atmosphere that oxidize the original gaseous components, such as sulfates and so on [43]. Currently, there are three methods, which are mostly often used to analyze the sources of atmospheric particulate matters, which are source inventory method, source model (dispersion model) method and receptor model method. The receptor model method is the most commonly used method for source analysis of PM_2.5_ in China [44]. The receptor model includes chemical mass balance method (CMB), positive matrix factorization (PMF) method, factor analysis (FA) method, principal component analysis (PCA) method, multi-linear engine (ME2) method and UNMIX method (UNMIX is a principal component method, but is based on geometrical analysis of the measurement dataset) [45]. Table 2 summarized the researches that have been conducted by the Chinese scholars on PM_2.5_ sources analysis. Some scholars also integrated these basic models with other methods for PM_2.5_ source apportionment analysis. For example, Wang et al. [46] used PMF model to derive PM_2.5_ contribution sources, and then used backward trajectory model to identify four potential directions to identify PM_2.5_ contribution sources, which shows that there was a clear difference in the distribution rates among all the different sources at different directions.

Shi et al. [70] employed a chemical mass balance gas constraint-Iteration (CMBGC-Iteration) method for source appointment analysis in Tianjin. The outcomes from this method were compared with the ensemble-average outcomes of CMB, CMB-Iteration, CMB-GC, PMF, WALSPMF (Weighted Alternating Least Squares Positive Matrix Factorization), and NCAPCA (Non-negative Constrained Absolutely Principle Analysis), and it was found that they were comparable. From Table 2, it can be found that the sampling time of most scholars is periodical, although sometimes with very long time span, the sampling work was done only in typical months of each season, or even a few days in a typical month. Although there are contingency and uncertainty with the measurements, it can still reflect the contribution source categories of PM_2.5_ to some extent. Meanwhile, it is observed that the contribution rates of different sources to PM_2.5_ from different scholars vary greatly, and there are obvious differences, even for the same city. It could due to the differences in the sampling time of the study, contribution categories, regions, climate, energy structure, atmospheric environment, etc.

### 2.2. Sources of Indoor PM_2.5_

**Figure 3:**
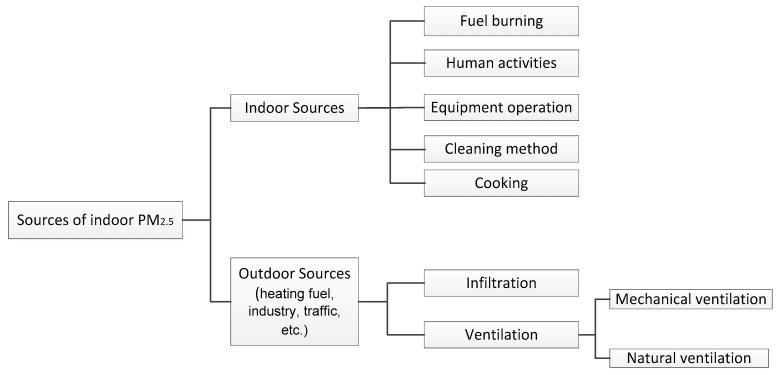
Sources of indoor PM_2.5_.

Sources of indoor PM_2.5_ can be divided into outdoor sources and indoor sources (see Figure 3). There is a time lag for the impact of outdoor PM_2.5_ concentration on the indoor PM_2.5_ to take effect. The indoor pollution sources are usually generated transiently and intermittently, resulting in large fluctuations in the concentration of indoor particulates [74].

#### 2.2.1. Outdoor Sources

The outdoor sources come from heating fuel, industry, traffic, etc. [34,46,47,48,49,50,51,52,53,54,55,56,57,58,59,60,61,62,63,64,65,66,67,68,69,70,71,72,73], due to the rapid industrialization, high energy consumption and large proportion of coal (60–70%) in the structure of energy sources in China. It has been acknowledged that there is close correlation between indoor and outdoor PM_2.5_ concentration levels, and outdoor PM_2.5_ is the main source of indoor PM_2.5_ pollution [75,76,77]. Wang et al. [78] showed that there was a significant correlation between indoor and outdoor concentrations of PM_2.5_ for rooms with normal airtightness and no air conditioning filter system. The indoor/outdoor (I/O) PM_2.5_ concentration ratio was up to 0.867. The correlation will be more obvious when the outdoor pollution level increases. Ji and Zhao [79] presented that 54–63% of indoor PM_2.5_ came from outdoors when the windows were closed, and it increased to as high as 92% when the windows were open. Han et al. [75] concluded that indoor PM_2.5_ concentration is significantly correlated with outdoor PM_2.5_ concentration but with 1 to 2 h delay, and the differences in the time lag effect are due to differences in environmental meteorological conditions such as outdoor air temperature, humidity ratio and wind direction.

#### 2.2.2. Indoor Sources

There are many different types of indoor PM_2.5_ sources, which mainly come from fuel combustion, human activities, equipment operation, cleaning, and cooking. Indoor combustion of fuels such as coal, natural gas, alcohol, and mosquito coils can lead to the rapid increase of indoor PM_2.5_ concentration. Zhang and Duan [80] showed that burning a mosquito coil ring could release 626 μg·m^−3^ of PM_2.5_, which is 8.3 times the concentration limit allowed for the residential environment. Li et al. [81] concluded that PM_2.5_ concentration in households using coal to cook was significantly higher than those using gas or electricity, and if coal is switched to gas or electricity, the PM_2.5_ concentration in the kitchen could be reduced by 40–70%. Zhou et al. [82] indicated that human activities such as walking, dressing and cleaning could result in increased indoor PM_2.5_ concentration by 33%. Gui et al. [83] conducted experiments on dry-sweeping, wet-sweeping and air-dry sweeping in an office. The average indoor PM_2.5_ concentrations before cleaning were 47.3 μg·m^−3^, 40.6 μg·m^−3^ and 39.4 μg·m^−3^, respectively. The average indoor PM_2.5_ concentrations were 109.7 μg·m^−3^, 97.5 μg·m^−3^ and 43.3 μg·m^−3^ after cleaning. The average PM_2.5_ concentrations were increased by 2.3 times, 2.3 times and 1.1 times, respectively. Therefore, it is recommended to use wet sweeping under ventilated condition as much as possible. Sun et al. [84] found that the printer also plays a role in contributing to indoor PM_2.5_ concentration, and that PM_2.5_ released by printers with different performances was quite different. Zhang et al. [85] advised that different cooking habits, cooking methods, raw materials and even seasoning strongly influence the composition of particulate matters.

### 2.3. PM_2.5_ Reginal Variations

PM_2.5_ pollution has significant regional characteristics. The pollution conditions within or between regions is interrelated. PM_2.5_ pollution in one region is affected not only by local pollution sources but also outside regions to different levels of extent. A large number of studies have shown that PM_2.5_ pollution has regional transmission characteristics [86,87,88,89,90]. For example, the study from Xue et al. [86] showed that about 22%, 37%, 28%, and 14% of the annual average PM_2.5_ concentration in Jin-Jin-Ji, the Yangtze River Delta, Pearl River Delta and Chengdu-Chongqing city group were contributed by outside region, respectively. The contribution of PM_2.5_ concentration from outside region for Hainan, Shanghai, Jiangsu, Zhejiang, Jilin and Jiangxi were all higher than 45%. For Beijing, Tianjin and Shijiazhuang, the outside contributions accounted for 37%, 42% and 33%, respectively. At the same time, some scholars also found that the degree of PM_2.5_ pollution in the same region gradually weakened from the urban center to the suburbs [91,92,93,94]. Zhang et al. [92] analyzed PM_2.5_ concentration data from 13 monitoring sites in Xi’an from 1 January to 26 April 2013, and found that the PM_2.5_ concentration in this area decreased from west to east, which is consistent with the characteristics of altitude and wind direction. Analysis from Zhao et al. [93] on the characteristics of PM_2.5_ and PM_10_ pollution in Beijing showed that the concentrations of PM gradually increased from the northern mountain region to the southern plain areas. In the central urban area, the concentrations were higher in the western part than those in the eastern part. There were some differences on the PM_2.5_ concentration levels between urban and rural areas in some cases. Wang et al. [95] indicated that although there were differences in the degree of PM_2.5_ pollution between the urban and suburbs areas, their variation trends were basically the same, which means that the degree of pollution in the suburbs area was affected to some extent by the high PM_2.5_ concentration in the city center.

### 2.4. Impact of Meteological Factors on PM_2.5_ Variations

Meteorological factors can significantly affect PM_2.5_ mass concentration, which can help to reduce or aggravate the urban air pollution. Song et al. [96] found that during high temperature weather in summer, although PM_2.5_ mass concentration was 2 to 3 times higher than that of low temperature period, the high temperature weather was still helpful to the diffusion of pollutants. Zheng et al. [97] also indicated that the effect of rainfall on the removal of particulate matter was obvious. The average PM_2.5_ concentration decreased by 56.3% following the rainfall, and PM_2.5_ mass concentration was less than 60 μg·m^−3^ within 72 h after the rainfall. Within 1 h after the rainfall, the PM_2.5_ concentration level stayed almost unchanged, and it kept declining within the next 12 h. Jiang and Li [98] showed that there was a negative correlation between PM_2.5_ mass concentration and precipitation. Large mixed layer thickness and unstable atmospheric layer junction help to the reduction of PM_2.5_ mass concentration. In Nanjing, the PM_2.5_ mass concentration was relatively low under northeast and southwest wind conditions, and it also had a negative correlation with the wind speed. High humidity did not help with the reduction of PM_2.5_ mass concentration but would affect the visibility. Humidity ratio of 60–70% is a turning zone for PM_2.5_ pollution. Some other scholars also showed that wind speed, wind direction, atmospheric stability, air humidity, rainfall, etc. also have significant impacts on the diffusion, dilution, agglomeration and retention of PM_2.5_ [99,100,101,102]. In addition, the meteorological factors that are mainly related to PM_2.5_ concentrations in different cities also vary due to the differences in emission intensity and diffusion conditions of pollutants. Zhang et al. [103] found that the meteorological factors related to PM_2.5_ concentration during winter in Shijiazhuang were relative humidity and average wind speed; the main meteorological factors related to PM_2.5_ concentration in Xi’an were relative humidity, average wind speed and maximum sustained wind speed; the ones in Beijing are relative humidity, average daily temperature, average wind speed, maximum sustained wind speed and minimum temperature; the ones in Taiyuan were daily average temperature, relative humidity, average wind speed, maximum and minimum temperature, and maximum sustained wind speed; and the ones in Guangzhou were relative humidity, average wind speed, maximum temperature and rainfall.

## 3. Various Impacts of PM_2.5_ Pollution

### 3.1. Impacts on Atmospheric Visibility 

Visibility refers to the maximum distance that a person with normal eyesight can see clearly the contour of the target under the prevailing weather conditions, and it is an indicator on the transparency of the atmosphere. Some scholars pointed out that in recent years the atmospheric visibility in China has reduced sharply, which is closely related to the increase of the concentration of fine particulate matter (PM_2.5_) in the atmosphere [104,105]. Low-visibility weather has a significant impact on traffic, health, ecological landscape, etc. Visibility is also the most direct indicator of a city’s air quality [106]. At present, the study on the relationship between PM_2.5_ and atmospheric visibility mainly focuses on the statistical relationship among atmospheric visibility, PM_2.5_ concentration and meteorological factors. The results show that there is an obviously negative correlation between atmospheric visibility and PM_2.5_ mass concentration [107]. Some meteorological parameters such as relative humidity [106,108,109] also affect the relationship between PM_2.5_ and atmospheric visibility. For example, Hao et al. [108] pointed out that when the relative humidity was ≤19%, there was an obvious logarithmic relationship between PM_2.5_ mass concentration and atmospheric visibility; when the relative humidity was 20–29%, the relationship became exponential; and when the relative humidity was ≥30%, power relationship became obvious. However, Wang et al. [110] presented different viewpoints. They suggested that PM_2.5_ mass concentration is not related to atmospheric visibility. The reason why PM_2.5_ can affect the visibility is due to the difference in the chemical composition of PM_2.5_ in different seasons as well as difference in meteorological conditions.

### 3.2. Impacts on Regional Climate

The energy balance of the Earth-atmosphere system determines the state of the climate. In general, the energy balance of the Earth-atmosphere system is in dynamic equilibrium. However, if the balance is disturbed or destroyed, it causes the Earth climate to change [111]. There are direct and indirect impacts of PM_2.5_ on the climate. For the direct impact, the PM_2.5_ affects the earth-atmosphere radiation budget by scattering and absorption of solar radiation and ground longwave radiation. At the same time, PM_2.5_ can block the solar beams from reaching the Earth’s surface, and increase the optical density of the visible light, thus cutting down the solar energy that reaches the Earth’s surface. As a result, the ground temperature goes down and the temperature at high altitude rises. Zhang et al. [112] found that the aerosol optical depth (AOD) at 500 nm in North China reaches 0.60–1.00 during the pollution period, where the fine-mode particles contribute more than 90% to the aerosol extinction characteristics and the single-scattering albedo of the aerosol is lower than 0.88. Hu and Liu [113] pointed out that there is a negative correlation between PM_2.5_ concentration and total surface radiation. Especially at noon, the correlation coefficient can reach −0.62. In addition, in September and December, it was found that an increase of 1 μg·m^−3^ in PM_2.5_ concentration would cause a decrease in total radiation of 1.8 W/m^2^ and 0.5 W/m^2^, a drop of ground surface temperature by 0.11 °C and 0.02 °C, and a drop of air temperature by 0.03 °C and 0.01 °C. Wu et al. [114] studied the impact of PM_2.5_ on the urban heat island (UHI) and found that higher PM_2.5_ concentrations leads to lower UHI intensity, especially during the daytime and the UHI can be reduced by up to 1 K.

The changes in the concentration of particulate matter can affect the formation processes of cloud and rainfall, and indirectly affect climate change. In the formation of rain, it is necessary to have a nucleus of condensation in order to form raindrops from water vapor. Other than salt in the seawater, the sources of nucleus of condensation come from PM_2.5_. If there are too many particles, they may “eat away water”, so that the raindrops in the sky are not growing, then, drizzle and clear weather days will become less than before. On the other hand, the existence of PM_2.5_ might help to increase the number of condensation nuclei, so that possibility of rainfall will increase, and extreme rainstorm can even be produced. Therefore, in areas with heavy precipitation, PM_2.5_ may encourage precipitation and bring more rainfall; while in areas with little precipitation, it may help to reduce rainfall. Simulation from Gui et al. [115] showed that the increase of aerosol particulates in different regions of China resulted in decreased air temperatures at the height under 2 m, decreased humidity ratio and precipitation in most parts of eastern China. Yao et al. [116] studied air pollution in the Jing-Jin-Ji region and its impact on evapotranspiration (ET). They suggested that PM_2.5_ concentration has a significant negative effect on ET in most cities and that amount of water for agricultural irrigation could be reduced at high PM_2.5_ concentrations. In addition, PM_2.5_ can also aggravate or mitigate the acidification of rainwater in the pollution area, depending on the major components of the ions contained in PM_2.5_. Li and Zhang [117] sampled and analyzed data on precipitation and PM_2.5_ in Xi’an in 2011, and found that PM_2.5_ in Xi’an is acidic, which is in consistent with the pH value of the precipitation.

### 3.3. Impact on Human Health

As early as in the 1980s, a large number of epidemiological studies abroad have shown that PM_2.5_ has obvious side effects on human health [118,119,120,121]. The studies in China on the relationship between PM_2.5_ and human health also fully proved that PM_2.5_ can cause increases in the incidence of pulmonary heart disease [122], respiratory disease [123], cardiovascular disease [124], cancer [125,126] and other diseases, and even the death risk [127,128,129]. Long-term exposure to ambient PM_2.5_ might be an important risk factor of hypertension and is responsible for significant hypertension burden in adults in China [130,131], and it leads to reduced lung function [132,133]. PM_2.5_ is a risk factor for asthma [134,135], and it was related to the onset of children cough variant asthma by reducing immune regulation and ventilatory function [136]. PM_2.5_ exposures might affect reproductive health. Significantly decreased fertility rates by 2.0% per 10 μg·m^−3^ increment of PM_2.5_ were observed in [137]. Wu et al. [138] found that ambient PM exposure during sperm development adversely affects semen quality, in particular sperm concentration and count. However, Zhou et al. [139] argued that air PM_10_ and PM_10–2.5_ (2.5 ≤ cut sizes ≤ 10 µm) exposures, not PM_2.5_, are risk factors of semen quality. In addition, the indoor PM_2.5_ exposure levels were positively associated with skin aging manifestation, including score of pigment spots on forehead and wrinkle on upper lip [140]. PM_2.5_ may lead to induced DNA damage and cell cycle arrest in lung tumorigenesis [141]. Repeated exposure to PM_2.5_ induces vascular inflammation [142]. Measles incidence was found to be associated with exposure to ambient PM_2.5_ [143]. Significant associations between PM_2.5_ and acute coronary syndrome (ACS) have also been found in most studies [144]. PM_2.5_ may induce oxidative stress and inflammatory responses in human nasal epithelial cells, thereby leading to nasal inflammatory diseases [145]. Ambient PM_2.5_ concentrations were significantly associated with influenza-like-illness risk [146].

PM_2.5_ is associated with mortality. There are papers and reports on PM_2.5_ sources and associated mortality in China as part of the Global Burden of Disease (GBD). It was estimated that the global premature mortality by PM_2.5_ was at 3.15 million/year in 2010 with China being the leading country with about 1.33 million [147]. Lin et al. [148] found significant associations between PM_2.5_ daily exceedance concentration hours (DECH) and cardiovascular mortality (3.0–5.02% increase in mortality rate per 500 μg·m^−3^ increase in PM_2.5_). Health burden study by Song et al. [127] suggested that PM_2.5_ in 2015 contributed as much as 40.3% to total stroke deaths, 33.1% to acute lower respiratory infection (ALRI, <5 years) deaths, 26.8% to ischemic heart disease (IHD) deaths, 23.9% to lung cancer (LC) deaths, 18.7% to chronic obstructive pulmonary disease (COPD) deaths, 30.2% to total deaths combining IHD, stroke, COPD, and LC, 15.5% to all cause deaths. Electronic hospitalization summary reports derived from 26 major cities in China between 1 January 2014 and 31 December 2015 showed that PM_2.5_ had a negative impact on incidence of delirium, which is an independent risk factor for morbidity and mortality among older surgical adults [149]. The non-accidental mortality rate increases with exposure to extreme weather condition, especially hot dry synoptic weather types (SWT) and warm humid [150]. The effects of ambient air pollution and temperature triggered out-of-hospital coronary deaths (OHCDs) in China [151]. It was found that there is a spatial correlation between the mortality of respiratory diseases in Chinese provinces, corresponding to the spatial effect of PM_2.5_ pollutions [152].

Some scholars have conducted studies on specific groups of people. For example, Li et al. [153] studied the relationship between pregnant women’s exposure to PM_2.5_ and the birth weight of newborns. Cheng et al. [154] pointed out that exposure of pregnant women in the third trimester, especially half a month before delivery, to high concentrations of PM_2.5_, will lead to an increased risk of preterm birth. It might also be associated with low birth weight (LBW) and small for gestational age (SGA) [155]. Chen et al. [156] showed that the allergenicity in children is potentially related to the indoor PM_2.5_ component and its content by comparing the toxicity of cells of allergic and non-allergic children exposed to indoor PM_2.5_. Tu [157] pointed out there is an impact of PM_2.5_ in Nanchang on the increase in outpatient pediatric respiratory disease outbreaks, with a maximum cumulative lag effect of 5 days. A 10 μg·m^−3^ increase of PM_2.5_ concentration in the atmosphere resulted in 0.43% increase of respiratory disease outpatient visits. Ouyang et al. [158] found that the PM_2.5_ concentrations are positively correlated with pneumonia hospitalization number of children, and their effect on boys is more obvious than that in the girls. PM_2.5_ was independently associated with the risk of intensive care unit admission due to pneumonia (ICUp), and the maximum effect occurred at 3 to 4 days after exposure [159]. There were positive correlation between high concentrations of PM_2.5_ and increasing daily emergency room visits [160]. In addition, PM_2.5_ might also affect people’s mental health. When exposed in haze weather for a long time, people could easily become depressed; in severe cases depression might also be induced. Study from Jia et al. [161] indicated that PM_2.5_ exposure might negatively affect mood regulation and increase the risk of mental disorder.

### 3.4. Impact on Human Behavior

PM_2.5_ is considered to be the “culprit” that causes hazy weather. It is harmful to people’s health and at the same time has affected all aspects of people’s living conditions. First of all, to cope with the frequent smog weather, people pay more and more attention to the prevention of PM_2.5_ inhalation. Wearing an anti-haze mask has become a popular habit in China. Gu and Xie [162] pointed out that in haze weather residents would go out with anti-haze masks and would selectively adjust their outdoor activities or change their ways of transportation depending on the outside air conditions. Residents will change their window opening behavior, such as window opening time and size to prevent PM_2.5_ penetration into the room [163,164]. Of course, turning on anti-haze air conditioners is also a preferred option due to their ability to reduce the PM_2.5_ concentration while maintaining high level of indoor thermal comfort [165]. In addition, the hazy weather is disruptive to the effects of many scenic landscapes, and therefore many tourists will change their travel decisions during hazy weather [166].

## 4. Indoor and Outdoor PM_2.5_ Relationship

### 4.1. Current Indoor PM_2.5_ Pollution Status

According to a survey on the life style of the residents, people in China spent 85% of their time indoors, of which 50% of their time was spent inside the buildings. In particular, the elderly spent 90% of their time indoors, of which 76% of their time was in the residential buildings [167]. Therefore, an indoor environment with an acceptable indoor PM_2.5_ mass concentration level is an essential prerequisite for healthy living of the residents. At present, there are not many researches on indoor PM_2.5_ pollution in China. However, it can be concluded that existing PM_2.5_ pollution in China is very serious (Table 3). Compared with the daily average limit of PM_2.5_ level of 35 μg·m^−3^ based on China’s latest ambient air quality standard, the PM_2.5_ concentration level in almost all of the buildings in the cities studied in Table 3 exceed the limit. In some heavily polluted public spaces, the PM_2.5_ concentration can exceed the limit by more than five times.

### 4.2. Ease of Indoor Environment Contaminated by Outdoor PM_2.5_

Some researchers have suggested that outdoor PM_2.5_ can enter the room through three ways, including natural ventilation, mechanical ventilation and infiltration [168]. For natural ventilation, the outdoor PM_2.5_ was driven by wind pressure and thermal pressure into the interior of the building, and often there is no filter to remove the particle matters. For buildings with central air conditioning system, the fresh air was introduced by mechanical ventilation and go through air filters, however, the filters cannot removed all the particle matters and hence the PM_2.5_ can enter the interior environment. The infiltration is related to air tightness. Due to the existence of cracks in the building envelope, the outdoor PM_2.5_ will penetrate through the crack even when the doors and windows are fully closed. It is very important to evaluate how easily the indoor environment can be contaminated by outdoor PM_2.5_. Currently, the indoor and outdoor particle concentration ratio (I/O ratio) and penetration coefficient are considered as two important parameters to be used for evaluation.

#### 4.2.1. I/O Ratio

Most of the studies on I/O ratios were carried out based on field data measurement under natural ventilation or infiltration (see Table 4). From Table 4, it is found that the I/O ratios obtained by different researchers vary greatly. It could be due to the differences in the outdoor pollution level, outdoor weather conditions (outdoor wind speed, wind direction, temperature, humidity ratio, etc.), indoor sources of pollution, the conditions of the building envelope itself (the airtightness of the outer windows, the degree of sealing performance of the outer window with the wall, cracks over the wall, etc.), and the air changes per hour (ACH) [76,82,169,170,171,172,173,174,175]. Lin et al. [173] explored the difference of PM_2.5_ pollution in Wuhan and Guangzhou. Strong seasonal variation patterns were found, and PM_2.5_ pollution in Wuhan was more serious than that in Guangzhou. Through sampling data analysis, Zhou [174] found significant negative correlation among PM_2.5_ mass concentration, temperature, and wind speed. Significant positive correlation between PM_2.5_ mass concentration and relative humidity, and relatively weak relationship between PM_2.5_ mass concentration and atmospheric pressure were found. Wang et al. [175] found the indoor PM_2.5_ concentrations were affected by the outdoor PM_2.5_ concentration and the degree of air tightness of the outer windows. Under the same outdoor PM_2.5_ concentration, the outer windows with higher air-tightness were less prone to be affected by the outdoor PM_2.5_.

#### 4.2.2. Penetration Coefficient

Through surveys on people’s window opening behavior, it is found that 60% of the people select to close the window under haze weather condition to prevent the outdoor PM_2.5_ from entering the indoor environment [185]. In the case of closing the doors and windows, study on the penetration of PM_2.5_ through the envelope cracks becomes particularly important. Some researches advised that the outdoor PM_2.5_ entering the building envelope through the cracks is the process of “penetration” [168,185,186,187], where “penetration coefficient” is the decisive factor to evaluate the rate of PM_2.5_ entering the indoor. Many foreign scholars obtained the penetration coefficients of fine particles through experimental measurements [187,188,189], e.g., Thatcher et al. [188] found that the penetration coefficient is larger for smaller fine particles. Some scholars in China have also conducted researches on determining the penetration coefficient. Their studies mainly focus on some influencing factors that affect the penetration coefficient of fine particles, such as the height of the crack [190], the roughness of the inner surface of the crack [191,192], the indoor/outdoor pressure difference [192], crack geometry [193], ACH [194] and so on. Due to the limitations of available devices and testing conditions, only a small number of studies in China currently focus on studying the penetration coefficient of PM_2.5_ alone. Based on previous studies, Li [185] discussed on the outer window penetration coefficient of PM_2.5_ is affected by multiple factors, including particle size, indoor/outdoor pressure difference, air exchange rate, and geometry and surface roughness of cracks in the building envelopes.

## 5. Indoor PM_2.5_ Control

### 5.1. Air Filter and Air Conditioner Combination

Since the outdoor PM_2.5_ pollution cannot be gotten rid of in the short run, it is important to control the indoor PM_2.5_ pollution level to reduce its impact on occupants’ health. Some researches focused on selection of certain combination of air filters. For example, Cao et al. [195] developed indoor PM_2.5_ pollution control model under mechanical ventilation, and advised on how to select certain combination of air filters with different particulate removal efficiencies for a central AC system. Tu et al. [196] conducted test on filter efficiency based on particle sizing and counting method and PM_2.5_ weight filtration method for multiple air filters of different materials and different particle removal efficiencies under the same experimental conditions. The relationship between these two filtration efficiencies provides a preliminary basis for the selection of PM_2.5_ air filter for indoor air conditioning and ventilation system. Wang [197] studied the filtration performance of different grades of PM_2.5_ filters and proposed suitable filter combination schemes based on the PM_2.5_ pollution status in different regions. Comprehensive evaluations on the performance of different filter combination schemes were conducted, which could be used as references for the design of primary air conditioning system. Based on the principle of mass conservation, Lv [198] developed an indoor PM_2.5_ concentration model of the primary return air-conditioning system and studied the impact of the changes of the filtration efficiency and the fresh air flow rate on the indoor PM_2.5_ concentration, when the filters are installed in the primary air section, return air section and supply air section, respectively. The results from these researches can only be used for the primary air supply of the central air-conditioning system. It is worth mentioning that split air conditioner systems are installed in most of the residential buildings in China for indoor environment control. The split system is a ductless system. It has an outdoor unit and an indoor unit, where the inside (evaporative) heat exchanger is separated from the outside (condensing unit) heat exchanger. No fresh air systems are equipped. In general, the measures taken by the residents in China to deal with outdoor PM_2.5_ pollution are to fully close the doors and windows. Hence, no fresh air can be treated by air filters and PM_2.5_ can still penetrate through the cracks. It is far from enough to fight with PM_2.5_ pollution by simply closing the doors and windows, so how to maintain a healthy indoor environment for buildings with split air conditioning system remains a problem to be solved in China.

### 5.2. Development of New Material for Air Filters

Some researchers dedicated to the development of new filter materials. For example, Zhao et al. [199] reported that high efficiency and low resistance air purification materials made by electrospun polyvinylidene fluoride fiber (PVDF) doping with negative ion powders (NIPs) can have purification efficiency of up to 99.9%. Zhang et al. [200] utilized high-thermal-stability polyimide nanofibers to develop a highly effective polyimide nanofiber air filter. The efficiency of the filter to remove PM_2.5_ from automobile exhaust at high temperatures can reach 99.5%. Li et al. [201] developed a reusable polyethersulfone hollow fiber membrane with high permeability using single-dry-jet wet-spinning technology. These filter materials have a high PM_2.5_ capturing capacity, and if they can be widely used in air conditioning system, the burden to remove the indoor PM_2.5_ will be greatly alleviated. However, due to the high initial investment cost, it is unrealistic to widely adopt this kind of filters in China.

Other researchers developed filters that can be attached to the window to allow air to flow through to reduce the filtering cost. For example, Liu et al. [202] introduced a polyacrylonitrile transparent filter that captures the PM through controlling the surface chemistry and microstructure of the air filters. It allows natural, passive ventilation to pass through the window and can achieve removal efficiency of up to 98.69% at transmittance of ~77% in haze weather. Zhao et al. [203] reported slip-effect functional nanofibrous membranes with purification efficiency of 99.09% and transmittance of 77% with low air resistance of 29.5 Pa. Khalid et al. [204] reported a blow-spinning technique for large scale coating of nanofiber transparent air filter on window screen which achieved standard PM_2.5_ removal efficiency of >99% with 80% optical transparency. However, the study from Shi et al. [205] found that the mean value of harmonic average air exchange rate when the windows are open is far below the national standard. Therefore, more measures are needed to be taken to further reduce the filter resistance to enhance natural ventilation.

### 5.3. Anti-Haze Room Air Conditioners Available in the Market

Frequent episodes of hazy weather remind the residents of the importance and urgency to improve air quality, and it has become a driving force for the traditional air-conditioners to be upgraded with PM_2.5_ purification functiond. Table 5 lists some of the popular AC products from different air conditioner companies with PM_2.5_ purification function, which come from Midea, Haier, Panasonic, Gree and KELON. For example, the air conditioner (AC) products of Midea utilize a washable PM_2.5_ purification module with an electronic generator to create an electric field in the dust collection device to captured charged particles, which effectively removes PM_2.5_. The air conditioning products of Haier use visualization function to capture PM_2.5_. Each AC unit is equipped with a 5-color indicator. When indoor PM_2.5_ level exceeds the high limit, the indicator turns red and urge the occupants to turn on the PM_2.5_ removal function, and it becomes blue when the indoor PM_2.5_ level is back to normal. Panasonic air conditioners release negatively charged “nanoe-G” to be absorbed by PM_2.5_ in the air, through which PM_2.5_ is negatively charged and collected by electric field with high efficiency. The air conditioning products of Kelon use “three processes” (stripping technology, packaging technology and melting technology) for PM_2.5_ purification. The air-conditioners listed are residential models, effective in room size of 10–50 m^2^ (2650–7200 W).

## 6. Conclusions

The problem of PM_2.5_ pollution in China is severe. It has seriously threatened the health of the residents. As compared with the developed countries, studies on PM_2.5_ in China are still lagging behind. However, with the establishment of air quality standards for PM_2.5_ in recent years and development of PM_2.5_ monitoring stations nationwide in China, research on PM_2.5_ in China has gradually been enhanced. The studies on PM_2.5_ have increased exponentially, and more and more disciplines have got involved in the study on PM_2.5_, which are mainly related to PM_2.5_ source analysis, the impact of PM_2.5_ on human health, relationship between indoor and outdoor PM_2.5_ pollution levels and indoor PM_2.5_ control. It is worth mentioning that there are several measurement methods and small differences in the measurement results of PM_2.5_ concentration might be found. Most of the researches on I/O ratio and percentage of outdoor source contributions were based on the weighting method, which is a direct and reliable method. Generally speaking, the studies discussed throughout the paper will not be greatly affected with different measurement methods in various researches. There are still many shortcomings with current researches. For example, some studies only collected data in the typical month of the season or even a few days in a typical month. Although the data could be representative, they may also be incidental. Nowadays, depression, autism and other psychological diseases frequently occur, however, little research of PM_2.5_ impact on mental health can be found in China. Most of the researches on the indoor and outdoor pollution correlations are conducted only for a specific building, i.e., under specific physical and meteorological condition [75,76,77,78,79], for example, in a residential apartment [75], or in an office building [76]. Although many useful results have been obtained from these studies, they depend largely on the experimental conditions at the time when data were collected and cannot be applied to more general situations. In addition, although the environmental monitoring network of PM_2.5_ in the outdoor atmosphere is already established in China, the establishment of monitoring network on indoor air PM_2.5_, which is more closely related to human health, is still lagging behind. It may be due to the facts that the indoor PM_2.5_ pollution concentration limit is not clear in China. At the same time, the cost of creating household monitoring network is high, and it is difficult to carry out long time, standardized, PM_2.5_ monitoring and data collection indoor.

## Figures and Tables

**Table 1:** Implementation time table for each country/organization on PM_2.5_ concentration limit.

Country/Organization	Annual Average Limit (μg·m^−3^)	Daily Average Limit (μg·m^−3^)	Notes	Web References
USA-1	15	65	Established in 1997	[8]
USA-2	15	35	Established in 2006	
USA-3	15	12	Established in 2012	
Australia	8	25	Established in 2003, not enforced till now	[9]
WHO air quality goal (AQG)	10	25	Published in 2005, and the limit is mainly for developing countries	[10]
WHO transition target-1 (the most flexible limit)	35	75	Compared with AQG value, long-term exposure at these levels increases the risk of death by about 15%	
WHO transition target-2	25	50	Among other health benefits, exposures at this level reduce the risk of death by about 6% (2% to 11%) compared with transition target-1	
WHO transition target-3	15	37.5	This is the lowest level for long-term exposure to PM_2.5_, at which total mortality, cardiopulmonary disease mortality and lung cancer mortality will increase with over 95% confidence	
EU-1 (2010–2015)		25	Published in 2008, executed in 2010, and not allowed to go beyond the limit in 2015	[11]
EU-2 (2015–2020)		20	Not enforced until 2020	
Singapore (long term target)	10	25	Established in 2008	[12]
Singapore-1 (2008–2014)	15		Established in 2008	
Singapore-2 (2015–2020)	12	37.5	Established in 2015	
Japan	15	35	Established in 2009	[13]
India	40	60	Established in 2009	[14]
China level 1	15	35	Established in 2012, fully implemented in 2016	[15]
China level 2	35	75		

EU: European Union.

**Table 2:** Modeling methods and analysis on sources of PM_2.5_.

Method	Reference	Location	Sampling Time Period	Main Sources of PM_2.5_ and Their Contribution Rates
CMB	[34]	Ningbo	15–24 March 2010; 31 May–9 June 2010; 10–19 December 2010	Urban dust (20.42%), coal dust (14.37%) and vehicle exhaust (15.15%)
	[47]	Urumchi	19–30 January 2013	Urban dust (24.7%), coal dust (15.6%) and secondary particles (38.0%)
	[48]	Qizhou	September 2013; February–March 2014; May 2014	Dust (21–35%), secondary particles (25–26%) and vehicle exhaust (21–26%)
	[49]	Ningbo	25–31 January 2010; 31 May–6 June 2010; 10–16 October 2010	Urban dust (19.9%), coal dust (14.4%), secondary sulfate (16.9%), vehicle exhaust (15.2%), secondary nitrate (9.78%) and secondary organic carbon (8.85%)
	[50]	Tianjin	13–20 May 2010; 20–27 October 2010; 19–26 December 2010	Open source (urban dust, soil dust and construction cement dust, total contribution of 30%),Secondary particles (secondary sulfate, secondary nitrate and secondary carbon, total contribution of 28%), coal dust (19.6%) and vehicle exhaust (15.9%)
	[51]	Chongqing	6–28 February 2012; 6–28 August 2012; 19–27 October 2012; 7–29 December 2012	Secondary particles (30.1%) and moving source (27.9%)
	[52]	Beijing	August 2012–July 2013, continuous for 5 to 7 days per month	Secondary inorganic salts (36%), organic matter (20%), vehicle/fuel (16%), coal burning (15%), soil dust (6%) and others (7%)
	[53]	Xining	26 February–4 March 2014; 22–28 April 2014; 19–25 September 2014	Urban dust (26.24%), coal dust (14.5%), vehicle exhaust (12.8%), secondary sulphate (9.0%), biomass burning (6.6%), secondary nitrates (5.7%), steel dust (4.7%), construction dust (4.4%), soil dust (4.4%), food and beverage emissions (2.9%) and other unidentified sources (5.2%)
	[54]	Xingtai	24 February–15 March 2014; 22 April–19 May 2014; 15–28 July 2014	Coal dust (25%), secondary inorganic particles (sulfate and nitrate, 45%), vehicle exhaust (11%), dust (9%), soil dust (3%), construction and metallurgical dust (1%) and other unidentified sources (3%)
PMF	[55]	Wuhan	July 2011–February 2012	Vehicle sources (27.1%), secondary sulphates and nitrates (26.8%), manufacturing emissions (26.4%) and biomass combustion (19.6%)
	[56]	Chengdu	29 April–17 May 2009; 6 July–6 August 2009; 26 October–26 November 2009; 1–31 January 2010	Soil dust and raise dust (14.3%), biomass combustion (28.0%), vehicle sources (24.0%) and secondary nitrates/sulfates (31.3%)
	[46]	Shenzhen	January–December 2009	Secondary sulphate (30.0%), vehicle sources (26.9%), biomass combustion (9.8%) and secondary nitrates (9.3%)
	[57]	suburbs of Shanghai	23 December 2012–18 February 2014	Secondary aerosol (50.8%), fuel combustion (17.5%), biomass combustion/sea salt (17.2%), raise dust/construction dust (7.7%), and coal-burning/smelting dust (6.9%)
	[58]	North China	3 January–11 February 2014	Coal combustion (29.6%), biomass combustion (19.3%) and vehicle sources (15.9%)
	[59]	Lanzhou	Winter 2012 and summer 2013	Steel industry, secondary aerosols, coal combustion, power plants, vehicle emissions, crustal dust, and smelting industry contributed 7.1%, 33.0%, 28.7%, 3.12%, 8.8%, 13.3%, and 6.0%, respectively, in winter, and 6.7%, 14.8%, 3.1%, 3.4%, 25.2%, 11.6% and 35.2% in summer
	[60]	Chongqing	2012–2013	Secondary inorganic aerosols (37.5%), coal combustion (22.0%), other industrial pollution (17.5%), soil dust (11.0%), vehicular emission (9.8%) and metallurgical industry (2.2%)
	[61]	Yellow River Delta National Nature Reserve (YRDNNR)	January–November 2011	Secondary sulphate and nitrate (54.3%), biomass burning (15.8%), industry (10.7%), crustal matter (8.3%), vehicles (5.2%) and copper smelting (4.9%)
	[62]	Shanghai	October 2011–August 2012	Coal burning (30.5%), gasoline engine emission (29.0%), diesel engine emission (17.5%), air-surface exchange (11.9%) and biomass burning (11.1%)
	[63]	Zhengzhou	April 2011–December 2013	Coal burning (29%), vehicle (26%), dust (21%), secondary aerosols (17%) and biomass burning (4%)
	[64]	Qingshan District, Wuhan	15 November–28 December 2013	Traffic exhaust (28.60%), industry (27.10%), road dust (22%), coal combustion (13.20%) and building dust (9.5%)
FA	[65]	Beijing	16 January–28 February 2013	Industrial dust and human activities (40.3%), biomass combustion and building dust (27.0%), soil and wind induced dust (9.1%), fossil fuel sources (4.9%), electronic waste sources (4.8%) and regional migration sources (4.6%)
PCA	[66]	Hangdan	January, April, July and October 2015	Secondary aerosol source, transportation, fossil fuel and biomass burning (46.5%), soil and construction dust (19.5%), steel industry (19.5%) and transportation (9%)
	[67]	Hangdan	October 2012–January 2013	Industry and coal burning (33.3%), secondary aerosol and biomass burning (21.7%), vehicle (12.8%) and road dust (9.1%),
WRF/Chem+ observation data analysis	[68]	Guangzhou	January–December 2013	Moving sources (37.4%), industrial emissions (32.2%), electricity emissions (12.2%), residential emissions (6.6%) and others (11.6%)
PMF and backward trajectory model	[69]	Heze	13–22 August 2015; 21–30 October 2015; 14–23 January 2016 7–16 April 2016	Secondary inorganic salt (32.61%), vehicle emissions (22.60%), raise dust (19.64%), coal dust (16.25%) and construction cement dust (9.00%)
Chemical mass balance gas constraint-Iteration (CMBGC-Iteration)	[70]	Tianjin	April 2014–January 2015	Secondary sources (30%), crustal dust (25%), vehicle exhaust (16%), coal combustion (13%), SOC (7.6%) and cement dust (0.40%)
Ensemble-average of CMB, CMB-Iteration, CMB-GC, PMF, WALSPMF, and NCAPCA				Secondary sources (28%), crustal dust (20%), coal combustion (18%), vehicle exhaust (17%), SOC (11%) and cement dust (1.3%)
Community Multiscale Air Quality (CMAQ) model	[71]	25 Chinese provincial capitals and municipalities	2013	Power plants (8.7–12.7%), agriculture NH3 (9.5–12%), windblown dust (6.1–12.5%) and secondary organic aerosol (SOA) (5.4–15.5%)
Particle Induced X-ray Emission(PIXE), XRay Fluorescence (XRF), and PMF	[72]	Xigngzhen District, Beijing	19 May 2007–19 July 2013	Coal burning (29.2%), vehicle exhaust and waste incineration (26.2%), construction industry (23.3%), soil (15.4%) and industry with chlorine (5.9%)
Inventory-Chemical Mass Balance (I-CMB)	[73]	Beijing	2012	Coal (28.06%), vehicle (19.73%), dust (17.88%), industry (16.50%), food (3.43%) and plant (3.40%)

CMB: chemical mass balance method; PMF: positive matrix factorization; FA: factor analysis; PCA: principal component analysis; WRF: Weather Research and Forecasting; WALSPMF: Weighted Alternating Least Squares Positive Matrix Factorization; NCAPCA: Non-negative Constrained Absolutely Principle Analysis.

**Table 3:** Current status of indoor PM_2.5_ pollution.

Building Type	Sampling Location	Sampling Condition	Average Indoor PM_2.5_ Concentration (μg·m^−3^)	References	Times Exceeding Limit Set by Standard
Public place	Chongqing	Business hour	211 (68–468)	[176]	6.03
Public place	Ma’anshan	Business hour	133.73 (74.96–259.28)	[177]	3.82
Residential building	Lanzhou	Daily routine	Kitchen: 124.75 (48.14–279.25); Bedroom: 118.91 (38.34–367.62)	[178]	3.56; 3.40
Residential building	Nanjing	No cooking, no smoking	80 (47–113)	[179]	2.29
Hospital	Shenzhen	Business hour	36.71 (4.98–318.01)	[180]	1.05
Government agency	Tianjin	Business hour	71.0 (1–380)	[181]	2.03
Shopping mall	Beijing	Business hour	47 (9–253)	[182]	1.34
Market	Beijing	Business hour	56.21–61.36	[183]	1.61–2.25
Food court	Nanchang	Business hour	164 (38.03–492.73)	[184]	4.69

**Table 4:** I/O (indoor/outdoor) ratio under different ventilation modes.

Ventilation Mode	Reference	Sampling Time Period	Building Type	Impact Factors of I/O Ratio	Results
Natural ventilation	[169]	1 December 2013–28 February 2014	Residential building	Outdoor PM_2.5_ mass concentration level	When the outdoor PM_2.5_ concentration is in the ranges of 0–33 μg·m^−3^, 34–65 μg·m^−3^, 66–129 μg·m^−3^, and ≥130 μg·m^−3^, the I/O ratios are 1.75, 1.05, 0.76 and 0.63, respectively
	[170]	April–December 2015 (one week per month, except in July and August)	School	Outdoor PM_2.5_ mass concentration level, ACH, wind speed and outdoor air temperature	The time average I/O is 0.69. It varies in the range of 0.1–5.46. The I/O ratio decreases with the increases of outdoor PM_2.5_ mass concentration level
	[171]	09:00–18:00, 13–15 March 2014	Laboratory complex building	ACH	48.7–57.3% of the PM_2.5_ pollutants come from indoor sources and the I/O ratio varies 0.90–1.23
Infiltration	[172]	September 2013–August 2014	Office	Outdoor dry bulb temperature, relative humidity ratio and wind speed	The average ACH is 0.10 under mild weather, 0.22 when the wind speed is 1.6–3.4 m/s, 0.39 when the wind speed is 5.5–8.0 m/s. The corresponding I/O ratios are 0.43, 0.56 and 0.62, respectively
	[82]	Winter, 2014	Residential building	Indoor pollution sources	When the indoor PM_2.5_ concentration reached its peak value, the I/O ratio was 0.67–0.89
	[76]	June 2013–August 2013; December 2013–February 2014	Office	Seasonal changes, wind speed and relative humidity ratio	The indoor and outdoor PM_2.5_ concentrations in winter were higher than those in summer and the corresponding I/O ratios were also higher in winter than in summer

ACH: air changes per hour.

**Table 5:** Air conditioner with efficient PM_2.5_ purification function.

Brand	Type	Capacity (W)	Energy Grade	Main PM_2.5_ Removal Technology	PM_2.5_ Removal Efficiency
Panasonic	KFR-36GW/BpSJ1S	3600	3	PM_2.5_ air filter	84%
Haier	KFR-50LW/16UCP22AU1	5300	2	PET antibacterial and anti-mildew air filter	99%
Gree	KFR-26GW/(26571)FNBh-1	2650	1	Group filters with strong PM_2.5_ capturing ability, primary air filter and high efficiency air filter	≥97%
KELON	KFR-72LW/EFVEA2(2N01)	7200	2	Inhibitory fins that inhibit the growth of 99.9% bacteria	≥99%
Midea	KFR-35GW/BP3DN1Y-QA100	3500	1	Washable PM_2.5_ purifying module and dust collecting device	90%
